# Editorial: Cardiovascular engineering

**DOI:** 10.3389/fcvm.2022.1089794

**Published:** 2022-11-21

**Authors:** Caglar Ozturk, Marianne Schmid Daners, Xuanhe Zhao, Ellen T. Roche, Christopher T. Nguyen

**Affiliations:** ^1^Institute for Medical Engineering and Science, Massachusetts Institute of Technology, Cambridge, MA, United States; ^2^Product Development Group Zurich, Department of Mechanical and Process Engineering, ETH Zurich, Zurich, Switzerland; ^3^Department of Mechanical Engineering, Massachusetts Institute of Technology, Cambridge, MA, United States; ^4^Department of Civil and Environmental Engineering, Massachusetts Institute of Technology, Cambridge, MA, United States; ^5^Cardiovascular Innovation Research Center, Heart Vascular and Thoracic Institute, Cleveland Clinic, Cleveland, OH, United States; ^6^Cardiovascular Research Center, Massachusetts General Hospital, Charlestown, MA, United States; ^7^Martinos Center for Biomedical Imaging, Massachusetts General Hospital, Charlestown, MA, United States; ^8^Department of Diagnostic Radiology Imaging, Imaging Institute, Cleveland Clinic, Cleveland, OH, United States; ^9^Department of Cardiovascular and Metabolic Sciences, Lerner Research Institute, Cleveland Clinic, Cleveland, OH, United States

**Keywords:** cardiovascular engineering, cardiovascular disease, cardiovascular devices, computational modeling, cardiovascular biomechanics, cardiac imaging, heart failure

Cardiovascular disease remains the leading cause of death worldwide, despite significant advances in medical technology and in our knowledge of the disease and the treatment (1, 2). Over the last two decades, we have seen remarkable development in the fields of technology, healthcare, and the biological sciences. Since the cardiovascular field has progressed so rapidly, it is essential that each major innovation must function increasingly better than current technologies to have a long-lasting impact. The combination of exploding knowledge and recent advances in biology, medicine, imaging, and engineering have all helped foster a creative and unique environment for cardiovascular engineering.

Given the complexity of cardiovascular physiology, this field of study must encompass a wide range of research, from basic to translational, and aim to create diagnostic strategies, interventions, and biomedical devices that employ engineering principles and methods for cardiovascular disease. This special issue focuses on recent developments, reviews, and applications in cardiovascular engineering, incorporating topics such as mechanical assist devices, medical imaging, cardiovascular biomechanics, and biomaterials. In this editorial article, we provide a summary of the featured work in this issue.

Several groups focused on mechanical circulatory support (MCS) devices, such as ventricular assist devices (VADs) and total artificial hearts (TAHs) to advance existing knowledge and technological solutions for cardiovascular disease. Two papers shed novel light on the role of artificial pulsatility in continuous-flow support devices. Fang et al. assessed the impact of pulsatility using rotor speed modulation sequences in the HeartMate 3. The authors conducted a comprehensive computational fluid dynamics investigation on the washout performance with a particular focus on slow washout regions as an indicator of elevated risk of in-pump thrombus formation. The findings suggested that artificial pulsatility mostly enhances the washout of the flow separation region distal of the outflow cannula curvature. Furthermore, a physiological data-driven iterative learning controller has been introduced by Magkoutas et al.. Their controller effectively tracks predefined pump flow trajectories, and aims to generate a physiologically relevant, pulsatile, and treatment-driven response for continuous-flow VADs. This is a significant advancement over continuous-flow VADs and speed-based controllers, and opens up new opportunities in the management of continuous-flow VAD therapy since patient-specific preload sensitivity may be chosen directly based on clinical feedback.

Fox et al. have emphasized the need for effective therapeutic options for infants and children. Their research focused on developing a novel, continuous-flow, magnetically levitated TAH that may be used as a therapeutic option for high-risk pediatric patients with heart failure. This detailed work on a magnetically levitated prototype, which included computational analysis, *in vitro* hydraulic testing and hemolytic assessment revealed promising hydraulic performance characteristics, supporting the pulmonary and systemic circulation adequately in a hybrid design configuration.

Besides MCS, a number of groups demonstrated innovative preclinical disease models as a testbed to drive cardiovascular engineering design, testing, and validation. Malone et al. developed a new bench top mechanical circulatory loop (MCL) that can model the normal heart function and heart failure with preserved ejection fraction (HFpEF)—a multifactorial type of heart failure with multiple phenotypes. Their MCL features two independently controlled cardiac chambers which can be altered to limit diastolic filling to mimic the severity of HFpEF and can accurately simulate the diastolic hemodynamics of the left atrium and left ventricle. In addition, a patient-specific lumped parameter model of HFpEF was developed and a prototype of a soft robotic extra-aortic counterpulsation device was created to investigate the physiological implications of varying operating conditions by Arduini et al.. Briefly, they investigated the feasibility of patient-specific testing of novel cardiac support devices using lumped parameter computational models based on clinical hemodynamic data. Although the advantages of counter-pulsation therapy are not yet totally elucidated, the framework provided in this study stimulate future research into the efficacy of counter-pulsation therapy as a treatment for patients with HFpEF.

Kolli et al. created an *in vitro* experimental flow loop employing patient-specific 3D-printed coronary arteries as a proxy for invasive cardiac catheterization laboratory measurements. In this pilot study, they evaluated coronary artery ischemia using an *in vitro* method, combination of non-invasive coronary CT angiography and 3D printing based fractional flow reserve (FFR). When compared to invasive FFR approach, their 3D printing based non-invasive environment showed a strong correlation and a consistency, paving the way for a non-invasive diagnostic technique that can eventually substitute invasive FFR.

Exarchos et al. describe research supporting the hypothesis that micro-structured surfaces with anisotropic topographies can enhance long-term endothelialization of cardiovascular implants (CVIs). Describing recent efforts to modify the surfaces of various CVIs, including stents, grafts, valves, and VADs, they discuss the associated advances and challenges that have been encountered during their clinical translation. In their original research article, the authors show that anisotropic topographies on micro-structured surfaces could restore the monolayer integrity of senescent endothelial cells, enhances cell alignment, and promote proliferation.

Regarding cardiac magnetic resonance imaging (MRI) approaches, a deep learning method for fully automatic strain analysis (termed DeepStrain) based on cine-MRI images was introduced by Morales et al.. The authors aimed to explore whether DeepStrain may identify asymptomatic dysfunction in young adults with cardiac risk factors such as obesity, hypertension, and type 2 diabetes mellitus. Reference strain values for asymptomatic LV diastolic and systolic dysfunction in various young adult populations have been presented using a fully automatic strain analysis software.

What's more, Huh et al. presented a novel 4D spatiotemporal reconstruction process for dynamically acquired single photon emission computed tomography data. Since cardiac deformation and respiration both reduce the quality of the reconstructed image, the authors developed an algorithm to reconstruct the dynamic sequence of independent respiratory and cardiac phases and tested it using the data generated from a Monte Carlo simulation.

In summary, this specific Research Topic on cardiovascular engineering provides a platform to exhibit a variety of original research, methods and review articles on recent scientific advances in cardiovascular implants, computational modeling, imaging, and *in vitro* simulators for pediatric and adult patients. We appreciate the efforts of all authors for helping make this such a fruitful special issue.

## Author contributions

CO drafted the Editorial manuscript. MSD, XZ, ETR, and CTN revised it for interpretation and content. All authors contributed to the article and approved the submitted version.

## Conflict of interest

The authors declare that the research was conducted in the absence of any commercial or financial relationships that could be construed as a potential conflict of interest.

## Publisher's note

All claims expressed in this article are solely those of the authors and do not necessarily represent those of their affiliated organizations, or those of the publisher, the editors and the reviewers. Any product that may be evaluated in this article, or claim that may be made by its manufacturer, is not guaranteed or endorsed by the publisher.
